# The First Case of Liver–Intestine En-Bloc Transplantation in Japan

**DOI:** 10.70352/scrj.cr.25-0340

**Published:** 2025-10-29

**Authors:** Hiroyuki Ogasawara, Kyohei Kasuda, Naruhito Takido, Ryusuke Saito, Yoshihiro Shono, Muneyuki Matsumura, Kengo Sasaki, Atsushi Fujio, Kazuaki Tokodai, Hironori Kudo, Michiaki Unno, Takashi Kamei, Motoshi Wada

**Affiliations:** Department of Surgery, Graduate School of Medicine, Tohoku University, Sendai, Miyagi, Japan

**Keywords:** composite liver–intestine transplantation, intestinal failure associated liver disease (IFALD), megacystis microcolon intestinal hypoperistalsis syndrome (MMIHS), multivisceral transplantation, pediatric transplantation

## Abstract

**INTRODUCTION:**

Simultaneous liver–intestine transplantation is indicated for intestinal failure associated liver disease (IFALD), which can be caused by conditions such as intestinal motility disorders or short bowel syndrome. Currently, the most common grafts for liver–intestine transplantation are multivisceral transplantation (MVT) grafts consisting of the liver, stomach, duodenum, pancreas, small intestine, and colon, or liver–intestine grafts derived from the MVT graft but without the stomach. However, at the time of transplantation in the present case, Japanese regulations did not permit simultaneous pancreas transplantation in a non-diabetic recipient, which is generally required for this type of graft. Therefore, in the 2 previously reported domestic cases, the liver and intestine grafts were transplanted separately as non-composite grafts. Early in the development of MVT, liver–intestine grafts, excluding the stomach, duodenum, and pancreas, were used. We adopted this graft configuration for a 14-year-old female with megacystis microcolon intestinal hypoperistalsis syndrome (MMIHS) complicated by IFALD and performed, for the 1st time in Japan, a composite liver–intestine transplantation under existing regulations.

**CASE PRESENTATION:**

The patient was a 14-year-old female who was maintained on minimal enteral feeding via an intestinal stoma and parenteral nutrition because of MMIHS. Since approximately year X-7, her IFALD progressed, leading to portal hypertension with gastrointestinal bleeding and hypersplenism, which led to a significant decline in her quality of life. She was listed for deceased donor liver–intestine transplantation and a suitable donor became available in December X. The stomach, duodenum, and pancreas of the donor were resected *in situ*, and a descending aortic graft was interposed at the caudal side of the superior mesenteric artery; arterial anastomosis was performed on the recipient’s infrarenal abdominal aorta. The hepatic vein was anastomosed using the piggyback technique. Although a small amount of parenteral nutrition was still required because of colitis, the patient was gradually progressing toward complete enteral nutrition. No evidence of rejection was observed, and the patient was discharged.

**CONCLUSIONS:**

We report successful composite liver–intestine transplantation using a graft that excluded the stomach, duodenum, and pancreas, which led to good clinical outcomes.

## Abbreviations

HA
: hepatic artery
HV
: hepatic vein
IFALD
: intestinal failure associated liver disease
MELD
: model for End-Stage Liver Disease
MMIHS
: megacystis microcolon intestinal hypoperistalsis syndrome
MVT
: multivisceral transplantation
PV
: portal vein
SMA
: superior mesenteric artery
SMV
: superior mesenteric vein

## INTRODUCTION

Several combinations of multiple organ transplantations have been reported, depending on the underlying disease. Among these, simultaneous pancreas–kidney and liver–kidney transplantations are relatively common. By contrast, simultaneous liver–intestine transplantation is performed less frequently, with approximately 30–50 cases per year in the United States and 2 cases reported in Japan.^1)^ Indications for liver–intestine transplantation include IFALD caused by conditions such as intestinal motility disorders or short bowel syndrome, liver failure with portal vein thrombosis classified as Yerdel grade 4, abdominal trauma, and unresectable low-grade malignancies involving the mesenteric root.^2,3)^ MVT was 1st proposed by Starzl and Kaupp in the 1960s^4)^ and was clinically applied for the 1st time by Grant et al., Casavilla et al., and Starzl et al. in the late 1980s.^5–7)^ Graft configurations can vary but are generally classified as classic MVT grafts containing the liver, stomach, duodenum, pancreas, small intestine, and colon^7)^ and modified MVT grafts containing the stomach, duodenum, pancreas, small intestine, and colon, but excluding the liver.^8)^ Currently, most liver–intestine transplantations use a liver–intestine graft that excludes the stomach from the MVT graft.^9,10)^ However, this procedure requires simultaneous pancreas transplantation, which was only allowed for patients with diabetes in Japan at the time of this case. Therefore, 2 previously reported Japanese cases used a non-composite approach in which the liver and intestinal grafts were transplanted separately.^1)^ Historically, in the early days of MVT, a liver–intestine graft, excluding the stomach, duodenum, and pancreas from the MVT graft, was also utilized. Here, we report the case of a patient with MMIHS complicated by IFALD, who underwent a composite liver–intestine transplant under Japan’s existing regulations by omitting the stomach, duodenum, and pancreas from the graft.

## CASE PRESENTATION

The patient was born at 37 weeks via cesarean section. She presented with a giant bladder, intestinal obstruction without structural occlusion, and microcolon. Rectal biopsy confirmed no morphological abnormalities in the enteric nervous plexus; consequently, she was diagnosed with MMIHS. Oral feeding was impossible; therefore, she relied on minimal enteral nutrition and parenteral nutrition. Around X-7, IFALD began to progress, and she developed gastrointestinal bleeding caused by portal hypertension and pancytopenia resulting from hypersplenism, severely impairing her quality of life. She was listed for deceased donor intestinal transplantation in X-2. Upon deteriorating to decompensated liver cirrhosis and developing recurrent gastrointestinal bleeding secondary to portal hypertension, which became refractory to medical management in X-1, she was also listed for liver transplantation. In December X, a suitable deceased donor became available, and simultaneous liver–intestine transplantation was performed.

**Fig. 1:**
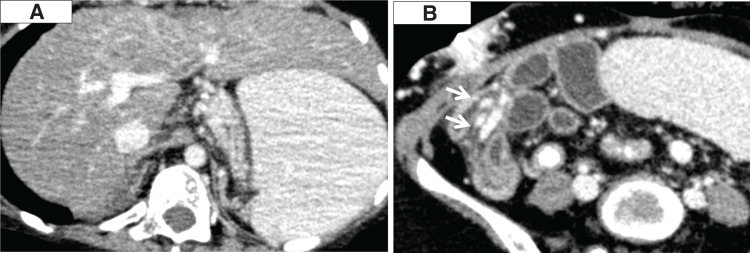
Pre-transplant CT images. (**A**) Liver cirrhosis and splenomegaly. (**B**) Prominent collateral circulation (arrows).

She was 14-year-old at the transplantation. Her preoperative height was 124.7 cm and body weight was 19.6 kg. As the Japanese deceased donor Liver Transplantation Eligibility Committee determined that she qualified for the point-addition system for IFALD, her MELD score at the time of registration for deceased donor liver transplantation was initiated at 16 points, with an increment of 2 points every 6 months. At the time a donor was allocated, her MELD score was 20. A total of 719 days had elapsed since registration for deceased donor intestinal transplantation and 464 days since registration for deceased donor liver transplantation. The donor was a teenage male with an intracranial hemorrhage. The donor/recipient body weight ratio was 138%. The donor/recipient body weight ratio was assumed to be acceptable within the range of 50%–100%. However, considering the deteriorating systemic condition of the recipient, prolongation of the waiting period was deemed unfeasible, and a slightly oversized graft was therefore accepted. The preoperative laboratory data of the recipient are shown in **Table 1**. Her Child–Pugh score was 9 (Grade B). CT revealed cirrhosis, splenomegaly, and a prominent collateral circulation (**Fig. 1**).

**Table 1:** Preoperative laboratory data

	Measured value	Reference value
White blood cell count, /μL	2100	3300–8600
Hemoglobin, g/dL	8.6	11.6–14.8
Platelet count, ×10^4^/μL	2.6	15.8–34.8
Prothrombin time, %	45.9	70–130
Active partial thromboplastin time, sec	52.9	24.3–34.6
International normalized ratio	1.49	<1.15
Total protein, g/dL	4.6	6.6–8.1
Albumin, g/dL	2.5	4.1–5.1
Total bilirubin, mg/dL	1.6	0.4–1.5
Aspartate aminotransferase, IU/L	57	13–30
Alanine transaminase, IU/L	26	7–23
Alkaline phosphatase, IU/L	120	38–113
γ-Glutamyl transpeptidase,IU/L	55	9–32
Blood urea nitrogen, mg/dL	14	8.0–20.0
Creatinine, mg/dL	0.44	0.46–0.79

### Donor procedure

[Fig F2][Fig F3]

**Fig. 2 F2:**
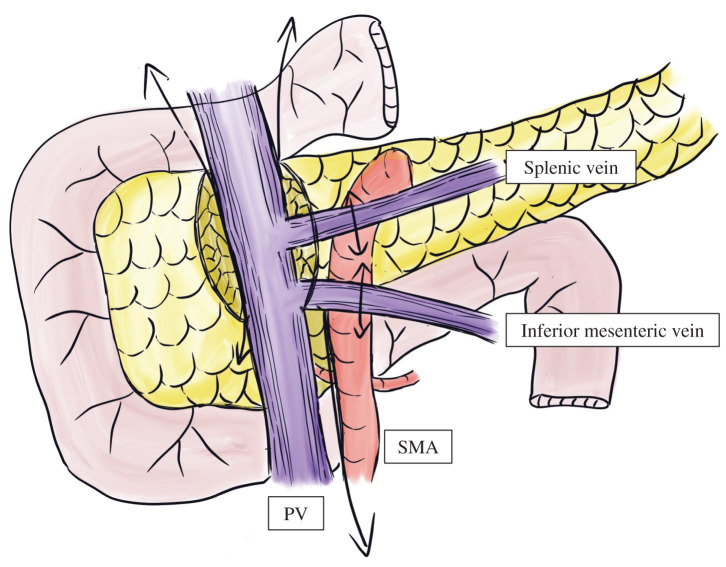
A technique for excluding the stomach, duodenum, and pancreas from the liver–intestine graft. The pancreatic head and duodenum were dissected en bloc using a pancreaticoduodenectomy-like technique, and the pancreatic tail and spleen were removed using a radical antegrade modular pancreatosplenectomy-like technique. PV, portal vein; SMA, superior mesenteric artery

**Fig. 3 F3:**
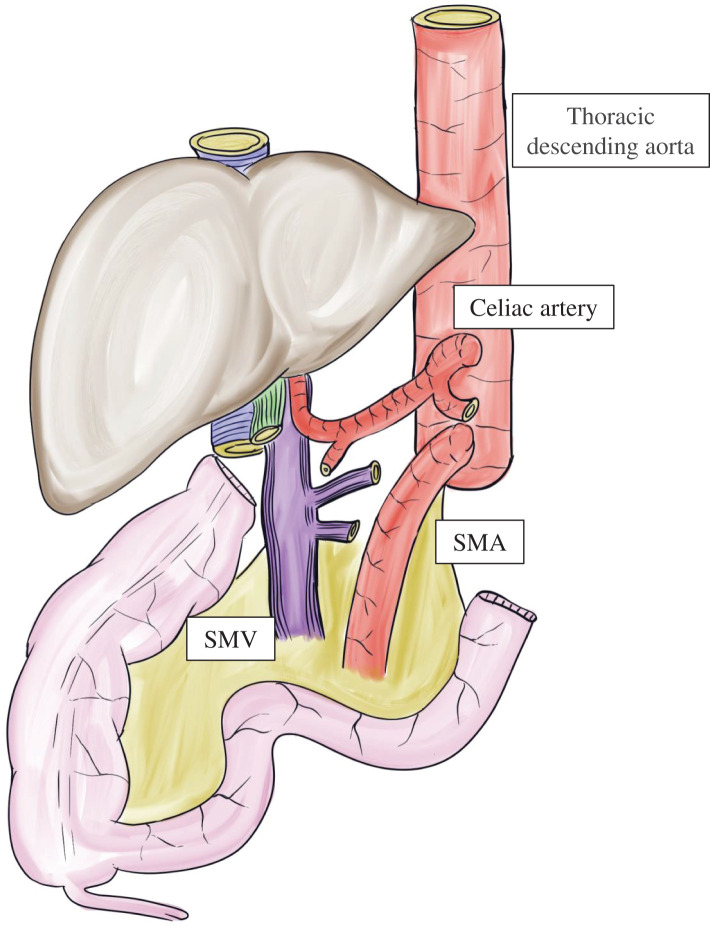
Liver–intestine graft at the time of procurement. The stomach, duodenum, and pancreas of the donor were resected *in situ*; the abdominal aorta and inferior vena cava were divided cranially to the renal vessels; and the graft was retrieved with the descending thoracic aorta attached. SMA, superior mesenteric artery; SMV, superior mesenteric vein

1. After cardiac and pulmonary grafts were procured, harvesting of the liver–intestine composite graft was initiated. The gallbladder was flushed, and the bile duct was divided at the superior border of the pancreas. The right and left gastric, gastroduodenal, and splenic arteries were divided down to the celiac trunks.
2. The stomach, jejunum, and transverse colon were divided following the full Kocher maneuver and colon mobilization.
3. The pancreatic head and duodenum were dissected en bloc using a pancreaticoduodenectomy-like technique, whereas the pancreatic tail and spleen were removed by a radical antegrade modular pancreatosplenectomy-like technique,^11)^ separating them from the PV and SMA (**Fig. 2**).
4. The abdominal aorta and inferior vena cava were divided cranially to the renal vessels, and the graft was retrieved with the descending thoracic aorta attached (**Fig. 3**). The time from aortic cross-clamping to graft retrieval was 92 min.

### Back table preparation

[Fig F4]

**Fig. 4 F4:**
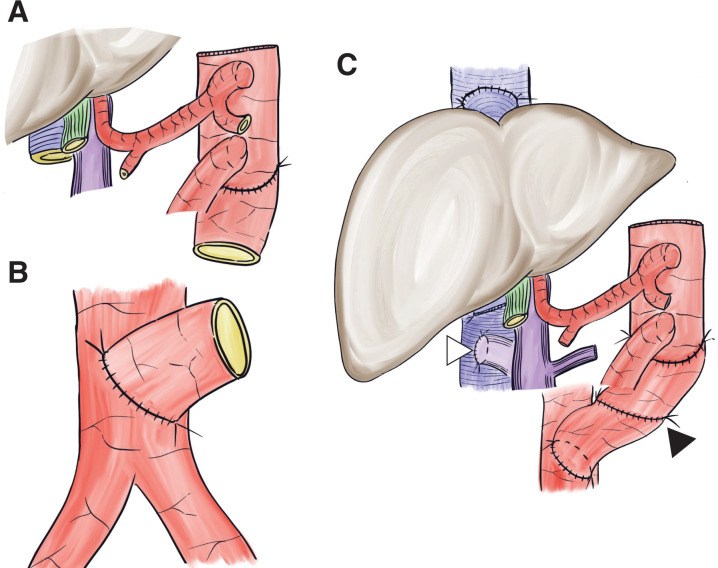
At the completion of vascular anastomosis. (**A**) The donor descending aortic graft was interposed at the caudal side of the graft’s superior mesenteric artery during back bench surgery. (**B**) A chimney-shaped vascular conduit was constructed on the recipient’s infrarenal abdominal aorta to facilitate vascular anastomosis using the same vascular graft. (**C**) The arterial inflow of the liver–intestine graft was then anastomosed to the distal end of the donor aortic graft (black arrowhead). Anastomosis of the hepatic veins was performed using the piggy-back technique. A portocaval shunt was created prior to graft implantation (white arrowhead).

1. The donor descending thoracic aortic graft was anastomosed to the caudal side of the SMA on the abdominal aorta to achieve arterial extension (**Fig. 4A**).
2. Meticulous ligation was performed around the SMV and the perivascular plexus of the celiac trunk and SMA.

### Recipient procedure

1. The patient had intestinal malrotation with no identifiable ligament of Treitz. The duodenum and sigmoid colon were transected and removed.
2. Total hepatectomy was performed, followed by the creation of a portocaval shunt. To secure sufficient abdominal cavity space, the spleen was removed.
3. The donor descending aortic graft was first anastomosed to the recipient’s infrarenal abdominal aorta (**Fig. 4B**). The arterial inflow of the liver–intestine graft was then anastomosed to the distal end of the donor aortic graft. Anastomosis of the HV was performed using the piggy-back technique (**Fig. 4C**).
4. After reperfusion, a choledochojejunostomy and intestinal reconstruction were performed. The Roux limb used for biliary anastomosis was connected to the recipient’s duodenum. An additional anastomosis was created between the elevated jejunum and recipient’s stomach, and the Roux loop was completed. A stoma was created in the transverse colon of the graft.
5. Open abdominal management was initially selected because of the difficulty of abdominal wall closure. The operation time was 17 h 14 min, and the total ischemic time was 9 h 29 min. The time from graft implantation to reperfusion (recipient WIT) was 47 min.

### Postoperative course

**Fig. 5:**
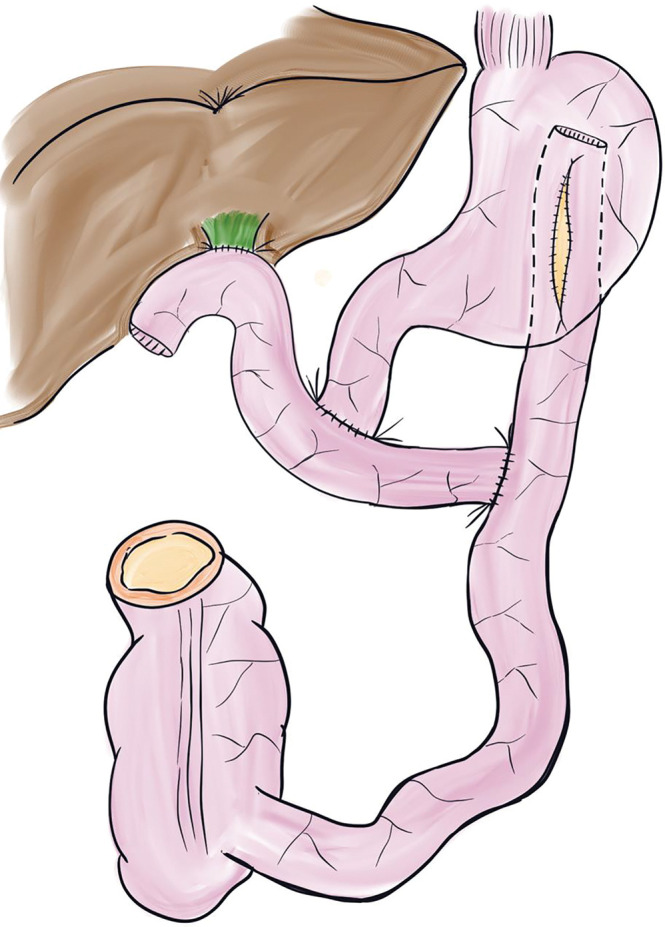
Intestinal reconstruction at the time of reoperation. A gastrojejunostomy was reconstructed on the posterior wall of the stomach, with the anastomotic orifice lengthened to improve outflow.

**Fig. 6:**
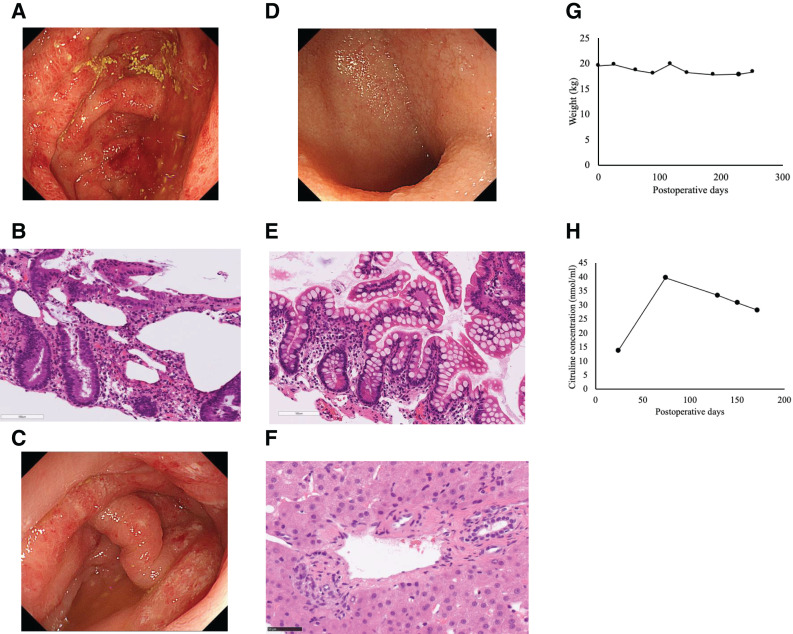
Postoperative course. (**A**) Inflammation of the colonic mucosa, predominantly involving the cecum, was observed. (**B**) There was mucosal inflammation with focal eosinophilic infiltration. (**C**) Pseudomembrane formation was observed in the colonic mucosa. (**D**, **E**) No inflammation or rejection was observed in the small intestinal mucosa. (**F**) No hepatic steatosis or rejection was observed. (**G**) Trend of body weight throughout the hospitalization. The patient’s body weight remained nearly stable during the hospital stay. (**H**) Trend of plasma citrulline concentrations throughout the hospitalization. Plasma citrulline levels improved and increased into the normal range.

On POD 4, abdominal wall closure was achieved, including fascial closure. The patient experienced transient acute kidney injury requiring continuous hemodiafiltration, which resolved spontaneously within several days (POD 7–9). Esophageal stricture developed as a complication of prior esophageal variceal treatment; therefore, balloon dilation of the esophagus was performed on POD 68. As sufficient energy absorption was achieved through enteral administration of nutritional formula, parenteral nutrition was discontinued on POD 88. Due to impaired gastric peristalsis associated with MMIHS, the outflow of saliva and gastric juice from the stomach into the intestinal graft was insufficient. On POD 130, a re-anastomosis was performed to enlarge the anastomotic site of the gastrojejunostomy (**Fig. 5**). Colitis developed on POD 157, and a biopsy was performed. No definite evidence of rejection was observed, and the patient was managed with careful observation. Because of impaired intestinal absorption, a small amount of parenteral nutrition was restarted on POD 165. On POD 171, eosinophilic colitis was suspected (**Fig. 6A** and **6B**), and treatment with increased doses of steroids and the initiation of a leukotriene receptor antagonist was undertaken. Subsequently, the patient developed pseudomembranous colitis on POD 218 (**Fig. 6C**), for which oral metronidazole was started. Thereafter, intestinal function gradually improved, and the patient was discharged on POD 262.

Immunosuppressive therapy included anti-thymocyte globulin, tacrolimus, and methylprednisolone. Multiple small bowel and colon biopsies were performed, but no evidence of rejection was observed (**Fig. 6D** and **6E**). In addition, a liver biopsy was carried out on POD 250, and no hepatic steatosis or rejection was observed (**Fig. 6F**).

During hospitalization, the patient’s body weight remained nearly stable (**Fig. 6G**). By contrast, plasma citrulline levels improved and increased into the normal range (**Fig. 6H**). Because gastric emptying into the intestinal graft did not improve, the patient was unable to achieve adequate oral intake. She was therefore discharged with a combination of enteral nutrition and a small amount of parenteral nutrition.

## DISCUSSION

Simultaneous liver–intestine transplantation is technically challenging and involves multiple organs; however, it can be a curative treatment for patients with severe complications such as intestinal failure and end-stage liver disease.^2,3)^ Grafts for liver–intestine transplantation can be broadly classified into 4 categories:

1. MVT graft (the liver, stomach, duodenum, pancreas, small intestine, and colon)^7)^
2. Liver–intestine graft excluding the stomach (the liver, duodenum, pancreas, small intestine, and colon)^9,10)^
3. Liver–intestine graft excluding the stomach, duodenum, and pancreas (the liver, small intestine, colon)^5,6)^
4. Non-composite grafts (the liver and intestine transplanted separately).

Although type 1 and 2 grafts are the most common worldwide, Japanese regulations at the time did not allow pancreas transplantation in patients who did not have diabetes. Consequently, the 2 previously reported domestic cases involved non-composite transplants.^1)^

In our case, we employed a composite liver–intestine graft by removing the stomach, duodenum, and pancreas, a technique originally developed by Grant et al., Casavilla et al., and Starzl et al. in the early era of MVT.^5–7)^ In the case of non-composite grafts, vascular anastomoses must be performed at 5 sites within the abdominal cavity: the HV, PV, HA, SMA, and SMV. Moreover, owing to underlying intestinal dysmotility and liver cirrhosis, the PV, HA, SMA, and SMV are often of small caliber, making these anastomoses technically demanding and highly complex. In the case of composite grafts, only the graft aorta (with a donor aortic conduit) and HV (via piggy-back) require anastomosis to the recipient. This approach avoids multiple small vessel anastomoses, potentially reducing the total ischemic time and warm ischemic time. In Japan, organ transport often takes longer because of the country’s geographical layout, making every effort to shorten the ischemic time especially critical. In liver–intestine transplantation, there are 2 approaches: simultaneous transplantation of both organs and a staged approach in which liver transplantation is performed first. The latter is an emergency strategy employed when a simultaneous transplant is not feasible because of a shortage of donor organs, whereby living-donorliver transplantation is performed initially, followed by waiting for a deceased-donor intestinal graft. Our institution has previously performed a heterochronic liver–intestine transplantation.^12)^ However, in some cases, IFALD may progress rapidly even after liver transplantation alone; therefore, simultaneous transplantation is preferable whenever possible.^13)^ Furthermore, simultaneous liver grafting may induce immunological tolerance, which can help attenuate rejection compared with isolated intestinal transplantation.^14,15)^ Compared with type 1 or 2 liver–intestinal grafts, the graft used in the present case did not include the pancreas, thereby avoiding the risk of post-transplant pancreatic complications such as pancreatitis.^9)^ Additionally, recipients of liver–intestine transplantation often have a limited intra-abdominal volume, which can make abdominal wall closure difficult.^16)^ In our case, resection of the stomach, duodenum, and pancreas reduced graft volume, facilitating abdominal closure. On the other hand, biliary reconstruction is required for this type of graft, which may increase the risk of biliary complication, such as bile leakage and cholangitis.^10)^ However, removal of the stomach, duodenum, and pancreas necessitates advanced hepatobiliary-pancreatic surgical skills and carries an increased risk of hemorrhage or vascular injury around the peripancreatic and perivascular plexuses. As donor availability increases and regulations continue to evolve, it may be safer and more straightforward to use a classic MVT graft or stomach-excluded liver-intestine graft. Indeed, in March 2025, Japanese transplant regulations were revised to allow simultaneous pancreas transplantation in liver–intestine transplant candidates, hopefully leading to more cases and improved outcomes. The type of graft used in the present case carries the risk of pancreatic resection; therefore, after the recent rule revision, the use of the MVT graft, which is more commonly employed overseas, would be considered more reasonable. However, in Japan, enough deceased donors are unlikely to be available for some time. Thus, situations may arise in which this graft type remains valuable—for example, when a slightly oversized donor relative to the donor/recipient weight ratio requires volume reduction, or when a donor has a benign pancreatic cystic lesion with a low but potential risk of malignant transformation. In such circumstances, the use of this graft could contribute to the expansion of the donor pool.

## CONCLUSIONS

We report successful composite liver–intestine transplantation using a graft that excluded the stomach, duodenum, and pancreas, which led to excellent clinical outcomes. Although this procedure was previously considered impractical under Japanese regulations, our experience demonstrates that it is feasible and safe with careful surgical planning and techniques. This case may help expand future indications and improve access to liver–intestine transplantation in Japan.

## DECLARATIONS

### Availability of data and materials

The datasets supporting the conclusions of this study are included in this article.

## References

[1] Okamoto T, Ogawa E, Okajima H, et al. Deceased donor non-composite split liver and intestinal transplantation for children. Surg Today 2025; 55: 716–22. 39168880 10.1007/s00595-024-02923-w

[2] Mangus RS, Tector AJ, Kubal CA, et al. Multivisceral transplantation: expanding indications and improving outcomes. J Gastrointest Surg 2013; 17: 179–87. 23070622 10.1007/s11605-012-2047-7

[3] Di Cocco P, Martinino A, Lian A, et al. Indications for multivisceral transplantation: a systematic review. Gastroenterol Clin North Am 2024; 53: 245–64. 38719376 10.1016/j.gtc.2024.01.007

[4] Starzl TE, Kaupp HA Jr. Mass homotransplantation of abdominal organs in dogs. Surg Forum 1960; 11: 28–30. 21566700 PMC3091286

[5] Grant D, Wall W, Mimeault R, et al. Successful small-bowel/liver transplantation. Lancet 1990; 335: 181–4. 1967664 10.1016/0140-6736(90)90275-a

[6] Casavilla A, Selby R, Abu-Elmagd K, et al. Logistics and technique for combined hepatic-intestinal retrieval. Ann Surg 1992; 216: 605–9. 1444653 10.1097/00000658-199211000-00014 PMC1242681

[7] Starzl TE, Todo S, Tzakis A, et al. The many faces of multivisceral transplantation. Surg Gynecol Obstet 1991; 172: 335–44. 2028370 PMC2655210

[8] Kato T, Nishida S, Levi D, et al. Multivisceral transplantation without the liver. Transplant Proc 2002; 34: 910. 12034233 10.1016/s0041-1345(02)02664-7

[9] Bueno J, Abu-Elmagd K, Mazariegos G, et al. Composite liver–small bowel allografts with preservation of donor duodenum and hepatic biliary system in children. J Pediatr Surg 2000; 35: 291–6. 10693683 10.1016/s0022-3468(00)90027-7

[10] Papachristou GI, Abu-Elmagd KM, Bond G, et al. Pancreaticobiliary complications after composite visceral transplantation: incidence, risk, and management strategies. Gastrointest Endosc 2011; 73: 1165–73. 21481866 10.1016/j.gie.2011.01.024

[11] Strasberg SM, Drebin JA, Linehan D. Radical antegrade modular pancreatosplenectomy. Surgery 2003; 133: 521–7. 12773980 10.1067/msy.2003.146

[12] Kudo H, Wada M, Sasaki H, et al. Intestinal transplantation at a single institution in Japan. Transplant Proc 2021; 53: 2040–5. 34266655 10.1016/j.transproceed.2021.06.021

[13] Ueno T, Wada M, Ogawa E, et al. Present state of intestinal transplantation in Japan. Pediatr Surg Int 2023; 39: 276. 37755555 10.1007/s00383-023-05552-5 PMC10533569

[14] Jugie M, Canioni D, Le Bihan C, et al. Study of the impact of liver transplantation on the outcome of intestinal grafts in children. Transplantation 2006; 81: 992–7. 16612274 10.1097/01.tp.0000195899.32734.83

[15] Abu-Elmagd KM, Wu G, Costa G, et al. Preformed and de novo donor specific antibodies in visceral transplantation: long-term outcome with special reference to the liver. Am J Transplant 2012; 12: 3047–60. 22947059 10.1111/j.1600-6143.2012.04237.x

[16] Fortunato AC, Pinheiro RS, Matsumoto CS, et al. Techniques for closing the abdominal wall in intestinal and multivisceral transplantation: a systematic review. Ann Transplant 2022; 27: e934595. 35228508 10.12659/AOT.934595 PMC8897964

